# 3D Tumor Models and Their Use for the Testing of Immunotherapies

**DOI:** 10.3389/fimmu.2020.603640

**Published:** 2020-12-10

**Authors:** Nicolas Boucherit, Laurent Gorvel, Daniel Olive

**Affiliations:** Cancer Research Center in Marseille, CRCM, Paoli Calmette Institute, Marseille, France

**Keywords:** spheroid, organoid, immunotherapy, tumor on a chip, tumor microenvironment, immune infiltrate, patient derived organoids, 3D culture

## Abstract

Over the past decade, immunotherapy has become a powerful and evident tool in the fight against cancers. Notably, the rise of checkpoint blockade using monoclonal antibodies (anti-CTLA4, anti-PD1) to avoid interaction between inhibitory molecules allowed the betterment of patient care. Indeed, immunotherapies led to increased overall survival in forms of cutaneous melanoma or lung cancer. However, the percentage of patients responding varies from 20 to 40% depending on the type of cancer and on the expression of the target molecules by the tumor. This is due to the tumor microenvironment which allows the acquisition of resistance mechanisms to immunotherapies by tumor cells. These are closely linked to the architecture and cellular composition of the tumor microenvironment. This one acts on different parameters such as the immune cells infiltrate its composition and therefore, favors the recruitment of immunosuppressive cells as well as the tumor expression of checkpoint inhibitors such as Programmed Death Ligand-1 (PD-L1). Therefore, the analysis and modeling of the complexity of the microenvironment is an important parameter to consider, not only in the search for new therapies but also for the identification and stratification of patients likely to respond to immunotherapy. This is why the use of 3D culture models, reflecting the architecture and cellular composition of a tumor, is essential in immuno-oncology studies. Nowadays, there are several 3-D culture methods such as spheroids and organoids, which are applicable to immuno-oncology. In this review we evaluate 3D culture models as tools for the development of treatments in the field of immuno-oncology.

## Introduction: Tumor Microenvironment and Immunotherapies

The tumor microenvironment (TME) represents tissue, cellular, and soluble factors which are being affected by the development or the evolution of a tumor. The TME affects the main function of the tissue such as its metabolism and vascularization as well as the immune system (1). The immune system in the TME proved to be a keystone of the tumor development. Indeed, the immune system is affected by the tumor at two levels. First, tissue resident immune cells are affected and see their phenotype and function modified toward tumor promoting profile. Second, recruited immune cells are either affected by the TME when they reach the tumor invaded tissue or at a distant site such as tumor-cell invaded draining lymph nodes. This will profoundly influence the becoming of the tumor, as it might be eradicated or might progress and metastasize (2). In the TME, immune cells are polarized to promote tumor growth according diverse mechanisms. TME metabolic constraints are known to increase myeloid-derived suppressive cells (3) (MDSCs) and regulatory T cells (Tregs) recruitment (4), as well as increasing inhibitory checkpoint molecule expression on immune cells such as PD-1, Programmed Death ligand-1 (PD-L1) (5) and CD47 a receptor part of the “don’t eat me signal” which avoids phagocytosis of tumor cells (6). Tumor cells such as CAFs (cancer associated fibroblasts) are known to limit the entry of anti-tumoral T cells (7) and to promote Tumor associated M2 Macrophages (M2) (8). Therefore, the TME affects every aspects of tissue homeostasis which explains why conventional treatments such as chemotherapy, radiotherapy or surgical resection (when possible) often leads to relapse in most aggressive forms of cancer (9). Recently the study of the immune system within the TME allowed to develop new treatments based on the targeting of inhibitory receptors present on tumor infiltrating leukocytes (CTLA-4, PD-1), and later on their ligands which are expressed by other immune cells as well as tumor cells (PD-L1) (10). Nowadays, more and more targets are being tested using mAbs, such as ICOS and TIGIT (11, 12). Also, asides of immune checkpoint receptors, soluble molecules are being targeted by mAbs. Indeed, cytokines such as TNF-α, IL-17, or IL-6 (13–15) or chemokines such as CCR5 and CXCR2 (16, 17) are being tested using several approaches and often in combination with checkpoint inhibitors or conventional treatments. Another approach of immunotherapies is to stimulate immune cells with tumor antigens, or carcinogenic antigens to induce a repertoire of immune cells which will only target the tumor. Indeed, the cancer vaccination approach uses peptide-based approaches and select synthetic long peptides, neoantigens, and tumor lysates to stimulate antigen presentation and therefore expend tumor reactive clones of T cells. In viro-induced cancer (hepatocellular carcinomas, cervical cancer) viral peptides can be used to prime the immune system to avoid infection and therefore the development of tumors (Gardasil, Cervarix). Cell based immunotherapy relies on the selection, activation, and/or genetic modification of immune cell types to direct them against the tumor cells. Indeed, dendritic cells can be activated *in vitro* or pulsed with tumor antigens to be activated and specifically present the antigen to cytotoxic T cells and polarize them to kill tumor cells (18, 19). More recently, Chimeric-Antigen-Receptor T cells have been designed by genetic editing of T cell receptor and co-receptors to be aggressive against the tumor (20). The migration and survival of CAR T-cells can be improved by the addition of cytokines and chemokines such as IL-7 and CCL19 (21, 22). However, as efficient as some of these treatments might be, the resistance of patients to immunotherapy remains an issue. Therefore, the prediction of a situation where the patient will not respond to the treatment is a keystone to improve Immunotherapies. On one hand, predictive murine models often intertwine human tumors and a mix of human transferred immunity and murine innate immunity. this occurs in patient derived xenografts (PDX) where a patient tumor or tumor cells are transferred to an NGS mouse model which still possess components of the innate immune system of the host (mainly tissue resident myeloid cells). On the other hand, the testing on human tumors remain difficult because 2D cell culture/co-culture do not represent the whole TME. This is why the emerging use of 3D cell culture models for the testing of immunotherapies represent an elegant alternative.

## 3D Models

Two-dimensional culture models, which are based on the growth and proliferation of a monolayer of cells, do not allow to fully understand cell-cell and cell-extracellular matrix interactions. 3D culture models generate a polarization of cells with a basal and an apical pole, which induces genomic and protein alterations (23–27). The tissue microenvironment and the extracellular matrix are altered in the presence of a tumor. This translates by an alteration of oxygen, nutrients, metabolites distribution as well as cell proliferation and interactions. However, none of the 2D models are able to assess all these important parameters at once, and therefore fail to fully represent *in vivo* interactions. 3D models, such as melanoma-derived spheroids exhibit better immuno-modulatory, proliferation, and activation abilities than 2D cultures (28).

**Figure 1:**
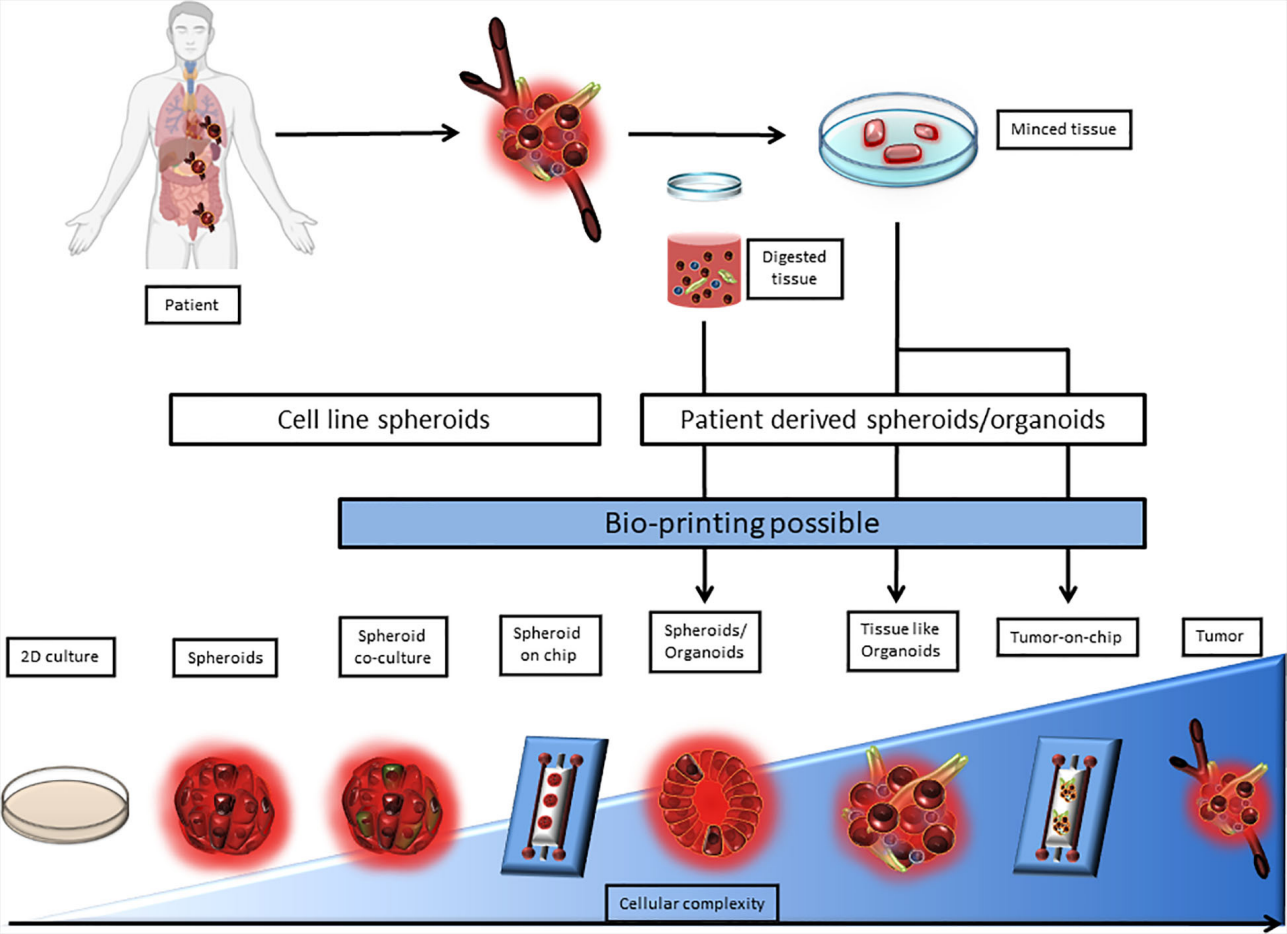
Representation of 3D culture models according to their complexity. 3D culture models are depicted as a range from spheroids derived from a single cell line to a very complex model derived from patient tissue or tumor upgraded with a microfluidic chip. 2D culture and tumor biopsy are used as complexity references. 3D cultures can be separated between cell line derived and patient derived models. Patient derived 3D models require either tissue mincing or both tissue mincing and enzymatic digestion prior to the culture. Noteworthy, Bioprinting can be used to generate most models that require multiple cell type-dependent structures, and can be applied directly on microfluidic chips.

The expression of immune checkpoint molecules *in vivo* differs from their expression in 2D culture models. This why it becomes critical to use 3D cell culture models that reproduce the TME in a more accurate way. Among 3D cell culture models, two terminologies are used, spheroids and organoids. In both of these models, technical advances allowed to complexify cocultures to better the TME representation. Indeed, Tumors-on-ship and bioprinting associate technology and 3D culture, to mime fluidics or tumor cell architecture. However, the difference between spheroid and organoids is blurry in terms of semantics and seem to be based on the author preference. The organoid term is often applied to healthy primary cells and tissue biopsy cultures, when tumor-organoid or spheroid are applied to cancer studies. Spheroid is used for simple 3D structures when tumor organoids is used for complex structures involving multiple cell types and miming tissue architecture. The border between the two terms still remains ambiguous especially when biopsies are used without any digestion step. Here, the term tumor-spheroid can be used when cell lines, digested biopsies, and non-digested biopsies cultured in non-adherent condition to generate 3D models. Tumor organoids should be used when tissue lysates or undigested tissue are cultured in an extracellular matrix to conserve the tissue architecture as well as the tissue diversity. According to this, we classified 3D models as a complexity gradient where cellular composition is at the center as shown in **Figure 1**.

### Cell-Lines Spheroids

The spherical model or spheroid model has been considered as the gold standard among the 3D *in vitro* models for the past 40–50 years. Spheroids are cells aggregates growing in suspension in three dimensions with or without an extracellular matrix. They have the ability to reproduce the architecture and metabolism of their tissue of origin to a certain extent. Indeed, they reproduce hypoxia (oxygen accumulation), nutrient gradient (glucose distribution), a necrotic/apoptotic core, lactate accumulation, and ATP distribution which the classical 2D culture failed to do (29). Several spheroids can be distinguished, based on cell origin and the culture methods: the multicellular tumor spheroid model (MTCS) using cells line and non-adherent support, tumorosphere using cells obtained from solid tumor dissociation, tissue-derived tumor spheres (TDTS) which comes from cells obtained by a partial dissociation of the solid tumor, and finally, organotypic multicellular spheroids (OMS), which differ of TDTS by the absence of tissue dissociation. Therefore, the methods to generate different type of spheroid vary (30). However, this classification remains blurry between the terms “spheroid” and “organoid,” especially when it comes to TDTS and OMS. Nevertheless, the term “spheroid” is commonly used to refer to cell line derived 3D cell cultures. The MTCS model, often derived from primary cell or cell line suspension is the most used and well characterized. Indeed, the MTCS model allows a good representation of oxygen, nutrient, and other soluble factor diffusion and exchange (31). However, to depict these parameters properly, the size of the spheroid needs to be comprised between 0.5 mm^3^ and 1 mm^3^ (29). A spheroid (>500um) is divided according to three areas from its core to its periphery. First, there is the necrotic/apoptotic core, then a quiescent cell layer, and, at the periphery, proliferative cells (32, 33) which mime tumor growth (30). It is nowadays the most used model for the assessment of immunotherapeutic strategies, thanks to its relatively low cost and high reproducibility (31).

Spheroid models may be used for the testing of immunotherapies, especially to assess the efficiency of therapeutic antibodies and drug screening for the enhancement of immune cell infiltration and anti-tumoral effects against the spheroid targets. Indeed, Courau et al. showed an NKG2D T cell and NK cell infiltrate with a colorectal cancer model of MTCS (HT29 cell line). The targeting of the NKG2D axis, and more precisely MICA/B molecules, highlighted an increase in NK cell infiltrate as well as a greater cytotoxicity. They also demonstrated that a combination of anti-MICA/B and anti NKG2A resulted in a synergistic effect against primary colorectal cancer-derived spheroids (34). Varesano et al. measured the anti-tumoral effect of Vd2 γδ-Tcells against colorectal carcinoma spheroids. Indeed, the authors showed the susceptibility to lysis of colorectal carcinoma spheroid subtypes by Vd2 γδ-Tcells, stimulated with zoledronate or cetuximab, by measuring physical characteristics of spheroids such as volume and area as well as their viability (35). MTCS models can be used to test the efficiency of CAR Tcells. Zhang et al. tested their mesothelin-targeting CAR T cells and found this treatment enhanced the anti-tumoral response in gastric and ovarian MTCS models (36).

Besides the generation of tumor-derived spheroids, another approach consists in the development of immune cell-derived spheroids. In an article where they generate J774.1 macrophage-derived spheroids in polydimethyl soloxane (PDMS) wells, Tanaka et al. could demonstrate that macrophage tend to polarize toward a tumoricidal M1 phenotype, by opposition to M2 pro-tumoral phenotype, in the spheroid condition (37). This seemed to be due to the hypoxia and the increasing production of reactive oxygen species, parameters which are generated by the structural properties of the macrophage spheroid. The authors proceeded to inject these M1 macrophage spheroids in insulinoma NIT-1 models, or colon adenocarcinoma, and they could demonstrate that spheroid injection led to greater biological activities compared to cell suspension spheroids (37). These models can also be improved and complexified by the technology and the systems being used. Indeed, in the MTCS scaffold-free monocellular model, Sherman et al. added a permeable layer on 96-well plates and could develop a 3D cell culture model which allows the screening of cell migration in a A549 lung carcinoma spheroid model (38). The authors highlight the fact that these models are limited by the absence of stromal cells, which are usually present in the tumor and are critical in the biology of the tumor as well as therapeutic resistance (38). Therefore, the possibility of increasing the diversity of cell types in MTCS cultures is enticing. Jeong et al. improved their colorectal carcinoma spheroid model by adding Cancer Associated Fibroblasts (CAFs). By doing so, they demonstrated that their spheroids became resistant to paclitaxel and modified protein expression, such as CD26 involved in the control of signal transduction for apoptosis and immune regulation (39). Hence, incrementing a monocellular model with another cell type seems necessary to strengthen the representation of the tumor microenvironment. However, to be accurate and mime the tumor composition, the new cell-type(s) should be introduced in the spheroid in a quantitatively accurate manner, meaning that cell ratios in the model should respect what is displayed by the tumor. This, requires an extensive study of tumor cellular composition before the creation of the model. In their scaffold-free MTCS model of pancreatic cancer, Lazzari et al. used the PANC-1 tumor cell line, along with MRC5 fibroblasts and the endothelial HUVEC cell line (40). In another example, Herter et al. demonstrated that the use of IgG-IL2v (an Immunocytokine) combined with the use of a tumor fibroblast-targeted T cell bispecific antibody (TCB) increased the immune infiltrate as well as their cytolytic function in their spheroid model (41). The presence of fibroblasts in the tumor cell culture allows and can even be necessary to allow the formation of spheroids. In our hands, it was the case for MIAPACA and LNCaP pancreatic cancer cell line. The coculture of MIAPACA-derived spheroids with monocytes led to an increase in immunosuppressive cytokines and the polarization of monocytes into monocyte-derived suppressor cells (MDSCs) or M2 polarized macrophages (42).

### Patient Derived 3D Models

Organoids are mini-organs reconstituted and embedded in an extracellular matrix. They are obtained from mechanically dissociated or enzymatically digested primary tissue, and arise from stem or slightly differentiated cells. Organoids reproduce the architecture as well as the cellular compartment diversity and organization of the parent tissue, which allows a better modeling of the tissue functions (43). Organoid culture appeared recently in the field of cancer research as the culture of tumor-organoids/Patient-derived organoids (PDOs) only started a decade ago. PDOs allow the 3D culture of cancerous cells isolated from primary tissue digestion, which leads to the loss of stromal and immune compartments. After growing the PDO, which is time consuming (2 to 3 weeks) (43), peripheral blood mononuclear cells (PBMCs) or other immune cell subsets can be added as a coculture. Among patient-derived 3D models can be found multiple models such as, but not limited to:

**Tumorospheres:** They are spheroid/organoid models, which are generated from cell suspensions after digestion of the original tissue of the tumor. These tumorospheres usually arise from cancer stem cells (CSC), where one isolated CSC should create a spheroid simply by proliferating. Therefore, tumorospheres are clonal models of spheroids/organoids. However, this spheroid model is limited to CSC study as it fails to reproduce the multiplicity of cell types in the TME, and is poorly reproducible as some CSC remain undifferentiated (44).

**Tissue derived tumor spheres (TDTSs):** They are obtained from enzymatic digestion of the original tumor tissue. This model is therefore composed of tumor cells only and allows to preserve tumoral cell interactions. Indeed, tumor cell interaction are rather strong and resist to enzymatic dissociation while stromal-to-tumor cell interaction are cleaved. TDTSs reproduce small versions of unvascularized tumor areas (30). Among TDTSs, there are spheroids and organoids models. Unfortunately, these denominations also vary with authors and therefore makes it harder to stratify the different models. TDTSs are often use in the study of colorectal tumors, and gave birth to different models such as colospheres. Colospheres are derived from colorectal cancer tumors, which were implanted as PDX in nude female mice and expanded. Tumors were then extracted from mice and cultured to form spheroids (45, 46). Other TDTS models are derived from breast tumors such as MARY-X. MARY-X is a model of TDTS, which was derived from a single breast tumor minced and engrafted as a PDX (patient-derived xenograft) on nude mice. These PDXs were dissociated and mice components removed (99% human cells, “MARY-X shake”). These cells spontaneously form spheroids which are used for experiments (47). Di Liello et al. could demonstrate a reproduction of patient tumor response to chemotherapy by using a spheroid model derived from a non-small cell lung cancer biopsy. This model of spheroid was generated from the cell suspension from the tumor digestion culture on an extracellular matrix (Matrigel) (48). Although tissue digestion preserves tumor cell interaction, the architecture of the tissue is lost and the loss of stromal cells. To palliate to this effect, James et al. developed an organoid model where tumor tissues are digested and cultured in a dome of Matrigel, in a growth factor-enriched media. Simultaneously, CAFs are cultured to generate fibroblasts. These fibroblasts were phenotyped for the expression of Vimentin and were devoided of KRAS mutations (carried by tumor cells). The coculture between the organoids and the fibroblast led to an increased resistance to gemcitabine, therefore showing the importance of the association of stromal and tumor compartments. Furthermore, the authors showed that the addition of lymphocytes in the coculture led them to migrate towards organoids through the Matrigel (49).

**Organotypic multicellular spheroid/organoid (OMS):** They are simply derived from minced tumor explants cultured media without enzymatic or mechanical digestion, the latter being required only for longer cultures and passages. OMS are often referred to as tumor explants, tumor slices, PDE (patient derived explant) or organotypic tumor slice culture (TSC). OMS certainly represent the closest models to the parental tumor as they conserve the origin tissue architecture as well as its cellular heterogeneity (30, 50). OMS can usually be cultured for a week according to different methods. Indeed, they can be submerged in culture medium, or with an Air-Liquid Interface. Air-liquid interfaces can be created by putting the OMS in contact with the culture media through a matrix (Geltrex, Matrigel, collagen) or membrane, by entrapping the OMS within gelatin or collagen, which is then put in the culture medium (“sponge method”). OMS can be used for drug testing (51) and biomarker discovery (52) as they represent valid patient pre-clinical models (53, 54). Interestingly, PDEs can be implemented within a microfluidic platform (55) (please see the *Organ-on-Chip* section). Breast cancer tumor explants have been maintained during 7 days in standard culture conditions, and could be used to determine resistance or susceptibility to FAC treatment, which consists in a combination of 5-FU, Adriamicyn, and Cyclophosphamide. However the authors did not describe the immune compartment in their study (51). The immune system component of OMS was investigated in a pancreatic ductal adenocarcinoma culture slice model were the authors observed the presence of CD8^+^ T cells (CD3^+^ CD8^+^), Tregs (CD3^+^, FoxP3^+^), and macrophages (CD68^+^, CD163^+^, HLA-DR^+^) (56). Noteworthy, the pancreatic tumor slice model could be kept in culture for 6 days (56). Powley et al. showed encouraging results for the testing of Immunotherapies using OMS (called PDE). Indeed, they showed that the treatment of melanoma PDE with Nivolumab increased the distance between CD8^+^ effector T cells and Tregs, avoiding Treg mediated suppression of CD8^+^ T cells (52). This study is not the sole example of the use of OMS for Immunotherapy testing. OMS derived from pancreatic ductal carcinomas, endometrial cancer, and prostate cancer were used to study new approaches for checkpoint inhibitors and cytotoxic cell recruitment (54, 57, 58). Using models of colospheres generated from the HT29 and DLD-1 cell lines as well as a patient-derived CTO (colorectal tumor organoids) model, Liu et al. evaluated the diffusion and distribution of Cetuximab, a monoclonal antibody targeting the extracellular model of the epidermal growth factor (EGFR) by MALDI-MSI. CTOs are organoids derived from colorectal tumors. Briefly, tumor fragments are embedded in gelatin to keep tissue integrity and cultured in stem cell media. CTOs poorly retain immune cells usually and often require cocultures with immune cells (59). They could show that the diffusion and distribution of Cetuximab in 3D tumor models was similar to those occurring *in vivo* in previous studies (60). Organoids can be cultured in the presence of immune cells to assess their anti-tumoral activity as well as to test methods to stimulate them. Therefore, the adoptive transfer of autologous lymphocytes becomes an attractive strategy (61). Colorectal cancer (CRC)-derived and Non-small cell lung carcinoma (NSCLC)-derived organoids can be cultured in the presence of autologous circulating lymphocytes and IFN-γ to increase antigen presentation. They combined this approach with the use of an anti-PD-L1 mAb to avoid any suppressive effect of IFN-γ derived PD-L1 expression. Among class I MHC expressing CRC-derived organoids, half exhibited an increase of IFN-γ secretion along with an increase in CD107a expression (a surrogate marker for degranulation) in CD8^+^ T cells. Interestingly, among the responders, 50% did not show tumor specific CD8^+^ T cells in the blood. In four over five cases, CD8^+^ T cells activity against organoids was specific, as illustrated by the expression of CD137. Indeed, CD137 was expressed by T cells cultured with organoids in the presence or absence of IFN-γ, and was not expressed when cultured with healthy tissue organoids or in resting conditions. The cell product (CD137^+^ or expended) showed cytotoxicity against tumor organoids compared to healthy tissue organoids. This effect could be reversed, by adding an anti-MHC class I mAb in the co-culture (62–64).

**Patient-derived organoids (PDOs):** PDOs and OMS are both tumor or tissue fragments which are cultured in a dish. The difference between OMS and PDOs mainly relies the fact that OMS are a one-step enzyme-free culture while PDO culture requires two steps. Indeed, the first step is similar to OMS culture and preserves tissue integrity. The second step is the organoid expansion, which requires enzymatic dissociation and allows long-term culture but affects tissue integrity. PDOs reflect protein and gene expression from the biopsies they originated from. Multiple models can be found such as non-small cell lung cancer (NSCLC)-derived organoids, CCRC-derived organoids and melanoma-derived organoids, glioblastoma organoids (GBOs). NSCLC-derived organoids, CCRC-derived organoids, and melanoma-derived organoids are minced human tumors, which were cultured on an Air-Liquid Interface cell culture dish, fed with media routinely, and passaged every 2–4 weeks by dissociating PDOs with collagenase IV (65). GBOs are generated by simply culturing tumor slices without enzymatic nor mechanical digestion on an orbital shaker. They can be kept for 2 weeks in these conditions. For extended culture times (>1 month) GBOs are cut to smaller pieces and divided in different subcultures (66). Neal et al. demonstrated that the PD-1/PD-L1 axis was conserved in NSCLC-derived organoids. Noteworthily, their PDO was generated without enzymatic digestion at the first step, whereas different passages to allow longer culture times required enzymatic digestion (65). This protocol allowed the culture of 14 different types of tissue and mimed 28 diseases, with a high culture success rate (73% after a month). The conservation of architectural and cellular features was assessed by microscopy and the presence of the stroma was done by Vimentin and SMA expression. The immune compartment was also observed in these PDO models as CD3^+^CD8^+^ and CD3^+^CD4^+^ T lymphocytes as well as B cells, Natural killer cells and macrophages could be observed (65). More precisely, some of the organoid infiltrating T cells harbored an exhausted phenotype (LAG3^+^TIGIT^+^PD-1^+^TIM3^+^) which also could be observed in tumor biopsies. Furthermore, the lymphocyte infiltrate in PDOs conserves its diversity as the TCR clonal diversity was assessed in a comparison between seven different organoid cultures and a biopsy of human clear cell renal carcinoma. The most expanded clones in tumors were the same in the organoid cultures. However, the immune compartment tends to decrease overtime, and seems to disappear after a month of culture. This effect can be slowed by the addition of cytokines such as IL-2 in the culture media. Therefore, the resemblance of PDOs with their tumor of origin is critical as organoids can therefore be used as tools for the study of response to immunotherapies. Targeting the PD-1/PD-L1 axis in immune-oncology was already proven to be efficient. However, the prediction of the patient response to its treatment is a keystone in the process of improving immunotherapies and PDO cultures might be of help. Neal et al. also tested the response to nivolumab in nine NSCLC, eight CCRC, and three melanomas PDOs. They found that six over 20 PDOs responded to the treatment as T cells expressed higher levels of IFNG, PRF1, and/or GZMB transcripts, and CD8^+^ T cells proliferated. Among the six organoids that responded to nivolumab, three were from NSCLC, two were from ccRC, and one melanoma. Ten PDO were tested with nivolumab, anti-CD3, and anti-CD28 to allow T cell proliferation and activation. Two PDOs cultured responded to these conditions as tumor infiltrating lymphocytes increased their cytolytic function against tumor cells (65). In another study, Scognamiglio et al. used chordoma-derived organoids to assess the response to nivolumab (anti-PD-1 mAb). Chordoma tissue from each patient were digested and cultured on a Matrigel matrix to form the organoid, then anti-PD-1 or anti-PD-L1 mAbs were added for 24 h. They could observe that PD-L1 expressing PDOs were disrupted and lysed after treatment with Nivolumab, which allows to theoretically predict the response to this immunotherapy in patients (67). Immunotherapies are not limited to antagonistic or agonistic mAbs directed against checkpoint markers such as CTLA-4, PD-1, or PD-L1. Indeed, CAR-T cells are being developed for hematological malignancies and start to be tested against solid tumors. Therefore, Jacob et al. created a model of glioblastoma organoid against which they tested CAR-T cell function. These organoids (GBOs) have the same histological and transcriptomic properties as the tissue the originated from. Also, the cell heterogeneity in GBOs is similar to that of parental tissues and allow to maintain the microenvironment up to 2 weeks after the beginning of the culture. However, the main differences between parental tissue and GBOs are the transcripts related to blood and vasculature functions as well as immune related genes. Among the aspects that are conserved in GBOs, EGFRvIII (epidermal growth factor receptor vIII) is an important marker for glioblastoma progression. Thus, the authors designed EGFRvIII-specific CAR-T cells and performed a co-culture with GBOs. They observed that CAR-T cells were infiltrating and expending in GBOs. This was happening concomitantly to a reduction of EGFRvIII/EGFR ratio intensity and an increase of cleaved-Caspase 3 levels, showing the killing of EGFRvIII^+^ cells. However, EGFR^+^EGFRvIII^−^ cells remained in the culture after 3 days of co-culture with CAR-T cells, showing the specificity of the CAR-T cells, but also that they may not be sufficient to eradicate the tumor (66). Considering the complexity of the immune-evasion mechanisms by tumors, the prediction of patient response to a specific immunotherapy is key. Noteworthily, there are two registered clinical trials involving cancer organoids for immunotherapy (NCT03778814, NCT02718235). Overall, these results indicate that cancer organoid culture is a promising system to generate tumor-reactive T cells, to predict immunotherapy sensitivity, and to examine combination. Taken together, PDOs represent valid preclinical models in the era of personalized medicine (68).

As complex as these models are, they still lack one or multiple compartments to allow the best representation of the *in vivo* system. Indeed, the vascular system, and therefore the diffusion of drugs, cellular products, and their penetration inside the tumor, is missing in these models. This is being studied and the use of microfluidic systems and/or microchips for the improvement of organoid models is being assessed.

### Organ-on-Chip

The three-dimensional spheroid/organoid culture models offer the possibility of approaching the architecture and functionalities of the tissue from which they originate, and despite the advances which make it possible to consider part of the micro-environment such as stroma cells and the TILs (Tumor Infiltrating Leukocytes), it still lacks the dynamics of the environment found *in-vivo*. The strength of the recently applied microfluidic technology in the field of oncology is to combine the advantages of 3D culture in a controllable and dynamic environment. Microfluidics add to the production of spheroids/organoids makes it possible to overcome this default and to position 3D models in a physiologically dynamic environment, making it possible to investigate several parameters of carcinogenesis and to carry out drug screening and predict the response therapies. In a simple manner, the spheroid/organoid formed is placed in a microfluidic chip, the medium being perfused with the addition or not of therapeutic agents (69, 70). This technology can be used for classical cell line-derived spheroids, but also PDOs. Nguyen et al. reconstituted a HER2^+^ breast tumor from four cell lines, along with its microenvironment. They combined breast cancer, CAF, endothelial cell, and fibroblastic cell lines and cultured them on a micro-fluidic chip. Indeed, they cultured breast tumor cell line and PBMCs in both lateral chambers of the chip, when CAFs where only present in one of the chambers endothelial cells were cultured as a monolayer in the central chamber. Although this model does not use a reperfusion system, this created a flow of media from the lateral chambers through the central one, and mimed a circulation in the tissue. They used their model to evaluate the effect of Trastuzumab, an anti-HER2 mAb and could show that the ADCC effect of trastuzumab was highly reduced in the chamber containing the CAF. The authors concluded that CAFs were modulating the immune cell functions by reducing their contact time with tumor cell lines. This model allowed the testing of a therapeutic agent in a complex 3D system, which allows perfusion of soluble molecules (71).

PDOs can also be placed on fluidic microchips. In a study using the 40 to 100 µm fractions of the digested tissue, the authors could generate organoids which contained the TME as well as the immune cell populations (B cells, CD4^+^ and CD8^+^ T cells, myeloid cell subsets). They could also demonstrate that some T cells expressed immune checkpoint markers such as PD-1, CTLA-4, and TIM3. These organoids were place on a microfluidic chip to test an anti-PD-1 treatment and the eventuality of a resistance to this treatment. The authors could show that CCL19 and CXCL13, two chemokines involved in the recruitment of immune cells, were produced in the anti-PD-1 treated organoids. The cytokine secretion profile was assessed and revealed that the organoids which were treated by anti-PD-1 expressed the IPRES (innate PD-1 resistance) signature. This signature is a cocktail of cytokines which are expressed by patient with a shorter progression free survival. Therefore, this microchip organoid model allows to assess the resistance to checkpoint blockade resistance, and maybe allow a better distribution of these treatments to patients (72–74). Different models of microchip exist and all are adaptable to the need of studies. Initially, these chips were used to study tumor cells migration as well as macrophages (75), dendritic cells (76), PBMCs in general (77), and TILs (78). Indeed, Moore et al. used a model of multiplexed microfluidic perfusion named EVIDENT (*Ex-Vivo* Immuno-oncology Dynamic Environment for tumor biopsies). This model can hold up to 12 tumor tissue samples which can interact with their autologous perfused TILs. They could test multiple immune checkpoint inhibitors simultaneously and observe immune cell infiltration and cytotoxicity. In these conditions the organoids can be cultured for 5 days. To test the effect of anti-PD-1 blockade, the authors used murine tumors generated by subcutaneous injection of MC38 cell line. TILs were isolated and expended, and incubated with an anti-PD-1 overnight before the experiment in the microfluidic system. Here, they could demonstrate that TIL infiltration of organoids as well as cytotoxicity was increased in anti-PD-1 treatment conditions. They could replicate this result by using a human biopsy of NSCLC after treatment of the TILs with anti-PD-1 (78). The microfluidic technology is therefore a tool in the study of 3D culture models and immunotherapies. Indeed, as we discussed above, Immune checkpoint blockade and CAR-T cells can be investigated at the level of infiltration, immune checkpoint expression and cytotoxicity (72). Although this model is not standardized, and that the design is dependent on each team, microfluidic are promising and allow to make another step towards *in vitro*-preclinical models (39, 68).

### 3D Bioprinting

Bio-printing allows the reconstruction of 3D tissue by organizing drops on a cell culture treated surface. These drops contain both extracellular matrix as well as tumor cells. The advantage of this model is that the organization of the tissue can be fully designed, and drops can be hardened chemically or mechanically to obtain the desired tissue resistance. Bio-printing therefore allows a very precise in terms of tissue architecture and cellular placements. There are three types of bio-printers, droplet bio-printing, extrusion bio-printing, and laser bio-printing. Droplet bio-printing, is the most used technique in the pharmaceutical industry thanks to its high throughput yield (79–81). As an example, bioprinting was used to generate a breast cancer model using MDAMB-231 cells and RAW264.7 macrophages. Here, the aim was to study the two cell type interactions in an accurate representation of the TME (82). Although bioprinting is a very recent and still little used technology in the field of tumor immunology, it remains a promising candidate in the testing of immunotherapy strategies (83).

## Discussion

As we described, 3D cell culture is constantly evolving and offers more and more opportunities to use these models as pre-clinical tests for the screening of therapies, as well as personalized medicine with PDOs. It is important to keep in mind that the evolution of 3D culture models evolves toward a better representation of human or murine tissues and tumors *in vitro*. The TME, CAFs, and TILs play a critical role in the evolution of a tumor and its resistance to diverse treatments (38, 84, 85), such as CAFs which reduce the ADCC effect of Trastuzumab (70). Therefore, 3D models provide a mean to study the TME by incorporating it in spheroids and organoids.

Cell line-derived spheroids coculture with fibroblasts and immune cells is relatively cheap, reproducible, and might be used as a high throughput technology to test therapeutic mAbs or drugs and even cell therapies such as CAR-T cells (86). The effect of therapies on spheroids is measured by microscopy, mainly by assessing sphere volume, circularity, and cell viability. However, not all cell lines spontaneously form spheroids. Indeed, to be a valid model, spheroids should reach a sufficient size to form a central apoptotic or necrotic core and therefore a gradient of oxygen, nutrient and lactate accumulation (29, 32, 33). Another limitation of the extracellular matrix embedded-MCTS is the effect of Matrigel or Geltrex on immune cells. Both matrixes are generated from murine sarcoma and therefore are compose of great murine antigen amounts, which can activate immune cell subtypes such as CD4^+^ T cells (87). These limitations can be avoided by using synthetic or collagen extracellular matrix (88). Recently, the culture of tumoral tissue digestion products in an extracellular matrix and a growth factor enriched culture media allowed the creation of patient derived spheroids or organoids. This model gets more and more attention and is extensively being studied because they accurately represent the origin tissue properties, even at the genetic level where mutations are conserved (64, 67). Immune cell coculture with PDOs represent a valid model to evaluate the effect of immune checkpoint blockade, CAR-T cell infusion, to educate T cells to recognize tumor antigens and to predict patient response to these therapies. Although PDO represent another step toward a complete imitation of *in vivo* tissues, these models also have inconvenient. Indeed, their culture requires the bio banking of samples, which can be expensive, and the time of these cultures can be long, up to 2 months to obtain a stable culture. Furthermore, the culture of PDO also requires a non-synthetic or collagen extracellular matrix (89).

Noteworthy, the possibility to transfer 3D culture back to 2D culture exists. This was performed on canine bladder cancer organoids. The culture medium was modified so that cell from the organoid would migrate to the bottom of the flask and create what the authors called a 2.5 organoid. This culture method is less restrictive and expensive than that of the 3D models, and still shares the major compartments of the original tumor tissue (90). However, this model does not seem suitable to study immunology or immunotherapy as the immune compartment is lost. This highlight the fact that the conservation or incorporation of the immune system remains challenging (90). In the organoid or spheroid models that require tissue digestion, the incorporation of PBMCs at the time of the culture is possible but do not represent the profile of the TILs. On the contrary, PDOs, which are not or only partially digested, conserve the original TILs but do not represent the part of immune cell which are recruited from the blood. Another issue in the PDOs is the survival of immune cell which can be boosted (up to a month) by adding IL-2 in the culture media. This ALI model allows the testing of Immune checkpoint blockade mAbs since the PD-1/PD-L1 axis is conserved. Furthermore, these PDOs can be bio-banked to allow further testing patient per patient. The major drawback of this model is that the first organoid culture has to be performed on a high-quality fresh tissue sample, which means that the time between surgery and the beginning of the culture as to be as short as possible. This impacts the reproducibility as the quality of the sample decreases quickly over time in terms of architecture and cell population viability.

To complete these already complex models, technologies such as microfluidics or microchips allow to culture organoids or spheroids into dynamic models. Indeed, they mime vascularization, cell and soluble molecules such as antibodies diffusion (49). The EVIDENT technology from Bornstein et al. pushed the use of microfluidics forward as it allows the testing of multiple conditions at the same time on a single chip. Although this method requires the freezing of both TILs and tissues, it open the way to pre-clinical models of organoids where the testing of multiple conditions or drugs are required at the same time. Furthermore, the fact that the experiment is performed on the same microchip avoid batch effects between conditions (78). The EVIDENT approach is rather fast (2 weeks compared to 1–2 months) and is still able to mime the vascularization that to the microfluidics. The notions of time and speed are critical here, especially when this model might be used for the testing of therapies and/or the assessment of patient response to the treatments. They also provide a time advantage against patient derived xenografts (PDX, patient tumor engrafted on NGS mice), which take 3 to 5 months to be used.

PDX, on their side, possess the advantages of *in vivo* models with vascularization and allow the testing of virtually any drugs or treatment. However, these models are transient as the engrafted tumor and its TME are slowly being replaced by that of the murine (91). Also, the patient immune system is not engrafted on the host and therefore would require the use of humanized hosts. PDX remain long and requires constant care and an animal facility. However, it remains possible to engraft organoids to mice, resect them the organoids to put them back in culture. Interestingly, PDX can also be resected from mice to be used as basis for organoids or spheroids.

Overall, 3D models are a crucial tool in the development of new immunotherapy strategies (92). Indeed, evolved models such as PDOs, coupled with microfluidics or not, represent promising pre-clinical models to test patient response to therapies.

## Author Contributions

NB designed and wrote the manuscript. LG designed, wrote, and proofread the manuscript. DO funded and proofread the manuscript. All authors contributed to the article and approved the submitted version.

## Funding

DO team was labeled “Equipe FRM DEQ20180339209.” DO is Senior Scholar of the Institut Universitaire de France. LG is supported by a Fondation ARC experienced post-doc/young researcher fellowship (Aides individuelles—session de printemps 2018- Post-doctorat en France).

## Conflict of Interest

DO is a cofounder and shareholder of Imcheck Therapeutics, Emergence Therapeutics, and Alderaan Biotechnology.

The remaining authors declare that the research was conducted in the absence of any commercial or financial relationships that could be construed as a potential conflict of interest.

The handling editor declared a past co-authorship with one of the authors DO.
